# Dietary yeast affects preference and performance in *Drosophila suzukii*

**DOI:** 10.1007/s10340-017-0932-2

**Published:** 2017-11-03

**Authors:** Nathalie Bellutti, Andreas Gallmetzer, Gerd Innerebner, Silvia Schmidt, Roland Zelger, Elisabeth Helene Koschier

**Affiliations:** 1 Research Centre for Agriculture and Forestry Laimburg, Pfatten, South Tyrol Italy; 2 Division of Plant Protection, Department of Crop Sciences, University of Natural Resources and Life Sciences, Vienna, Austria

**Keywords:** Spotted wing drosophila, Life history, Egg-laying behaviour, *Hanseniaspora uvarum*, *Saccharomyces cerevisiae*

## Abstract

Yeasts play an important role in nutrition physiology and host attraction of many *Drosophila* species, and associations with various yeast species are documented for several drosophilid flies. The pest *Drosophila suzukii* (Matsumura) has a predominant association with the yeast *Hanseniaspora uvarum.* However, research has not been conducted on the nutritional physiology of the yeasts associated with *D. suzukii* (spotted wing drosophila). Therefore, in this study, we determined whether dietary yeast was nutritionally relevant and whether yeast species closely associated with *D. suzukii* positively affected life-history traits. Our results confirm a crucial role of dietary yeast in the larval development and survival of *D. suzukii.* Furthermore, we found specific effects of the closely associated yeast species *H. uvarum* and *Candida* sp. on larval survival. Observations of the egg-laying behaviour of *D. suzukii* on cherry fruits artificially colonised with different yeast species revealed that the number of eggs laid increased on fruits colonised with *Candida* sp. and *Saccharomyces cerevisiae*.

## Key message

- *Drosophila suzukii,* the spotted wing drosophila, is an invasive pest species that damages unwounded, healthy fruit.
- Although associations with yeasts have been previously documented, the nutritional effect of yeasts for this insect pest is unknown.
- Yeast was proven to be an essential nutritional source for larval development and affected adult oviposition performance.
- These findings can be useful for improving both attract-and-kill technologies and mass rearing of *D. suzukii*.

## Introduction

*Drosophila suzukii* (Matsumura) (Diptera: Drosophilidae), the spotted wing drosophila, is a highly polyphagous invasive pest native to Asia. Recently, the fly has spread throughout most of the principal fruit-growing areas of North America and Europe (Hauser 2011). Asplen et al. (2015) recently reviewed the invasion biology, its current global distribution and the economic effects of *D. suzukii*. Unlike most other drosophilid flies, which have a preference for overripe or fermenting fruit, *D. suzukii* damages unwounded, ripening fresh fruits with their sclerotised, serrated ovipositor (Kanzawa 1939). The fly reproduces and develops on a wide range of small and stone fruit crops and on both wild and cultivated forms. Soft-skinned and fleshy fruits are preferred (Walsh et al. 2011; Calabria et al. 2012; Kenis et al. 2016). The host attractiveness for oviposition and the host fruit suitability for larval development can differ widely among fruit species, cultivars and ripening stages. In several studies, raspberries and blackberries were the preferred hosts for oviposition (Lee et al. 2011; Bellamy et al. 2013; Burrack et al. 2013; Abraham et al. 2015; Diepenbrock et al. 2016). Most studies on host suitability focus on fruit characteristics such as sugar content, pH or fruit firmness (Burrack et al. 2013; Arnó et al. 2016; Lee et al. 2016). Hardin et al. (2015) showed that artificial diets with a low protein or carbohydrate content reduced the survival of *D. suzukii* larvae and prolonged their development time compared with the standard and fruit diets. Also, *D. suzukii* larvae developed more rapidly on ripe blueberries, a natural host, than on a protein-rich standard artificial media diet, although other fitness measures did not vary between the two diets (Jaramillo et al. 2015 and see Hamby et al. 2016 for a complete review). However, host preferences and suitability may also vary because of differences in the microbial community on the respective host fruits, with close associations between microbes and *Drosophila* previously well documented (Starmer 1981, 1982; Begon 1982; Chandler et al. 2012; Hamby et al. 2012). Yeasts associated with *Drosophila* are primarily in the phylum Ascomycota and the family Saccharomycetaceae (Starmer et al. 1990; Chandler et al. 2012; Hamby et al. 2012) and are a primary food source in the nutrition of adults and larvae of many *Drosophila* species (Bouletreau-Merle et al. 1978; Begon 1982; Becher et al. 2012). To date, the importance of yeast in the diet of *D. suzukii* has not been examined.

Studies on the nutritional importance of dietary yeast in *Drosophila melanogaster* (Meigen) demonstrate an explicit influence of specific yeast species on larval and adult fitness traits (Anagnostou et al. 2010a, b). In those experiments, the yeast *Metschnikowia pulcherrima* was less favourable for *D. melanogaster* survival, development time and adult body weight than other yeasts such as *Saccharomyces cerevisiae*. Additionally, the larvae and adults of *D. melanogaster* preferred different yeast species (Anagnostou et al. 2010b). Similar differences in the preferences of adult and immature *Drosophila* are also described in other studies (Cooper 1960; Fogleman et al. 1981). However, the specific effect of a single yeast species may not be the most important factor. According to Rohlfs and Kürschner (2010), an increase in species diversity and an appropriate combination of dietary yeast species have favourable effects on *D. melanogaster* fitness traits. Therefore, based on these findings, we assumed that specific yeast species might have similarly variable effects on *D. suzukii* life-history traits.

In this study, we examined the importance of dietary yeast as a nutrition source in the development of *D. suzukii*. We investigated the dietary effects of different yeast species on *D. suzukii* larval and adult fitness traits by evaluating the nutritional quality of respective yeast species on larval development and adult oviposition performance.

## Materials and methods

### Insects, diets and plant material

A laboratory population of *D. suzukii* was used for bioassays with larvae, whereas both a laboratory and a wild population were used for bioassays with adult flies to detect possible adaptations of laboratory-reared insects to dietary *S. cerevisiae* in the artificial rearing diet. The laboratory population was from various infested fruits collected in South Tyrol (province of Bolzano, Italy). It was maintained on *D. suzukii* cornmeal diet (DSCD) (Bellutti 2017) supplemented with dry baker’s yeast (*S. cerevisiae*, commercially available, Küchle GmbH & Co. KG, Günzburg, Germany) (DSCD + S.c.) under standard laboratory conditions (22 ± 0.5 °C, 75 ± 3% relative humidity, photoperiod L16:D8) for more than ten generations. The wild population originated from infested cherry fruits (*Prunus avium* subsp. *duracina* cultivar ‘Kordia’) in South Tyrol and was reared for one generation under the laboratory conditions described above on cherry fruits. More than 2000 field-collected *D. suzukii* individuals were used to establish both populations.

For the bioassays with larvae, the composition of ingredients of the DSCD was modified, and the resulting diets [DSCD(a)–(c)] varied in their nutritional quality (Table 1). To assess the effect of the yeast nutritional components on larval development, DSCD(a) was prepared without yeast and DSCD(b) without yeast and vitamins as a control for DSCD(a), whereas DSCD(c) was prepared without yeast and other protein sources. The ripe cherry fruits (cultivar ‘Kordia’) used in the experiments with female flies originated from orchards in South Tyrol. All bioassays were conducted under the standard laboratory conditions as described above.

**Table 1:** *Drosophila suzukii* cornmeal diet (DSCD) used to rear *D. suzukii* larvae. Variations in the nutrient composition are labelled as follows: DSCD(a), (b) and (c)

Ingredient	Trade mark	Quantity^a^	Diets			
			DSCD	DSCD(a)	DSCD(b)	DSCD(c)
			Full diet	Yeast-free	Low nutrient	Minimal nutrient
Distilled water		873.4 mL	x	x	x	x
Agar	Laboratorio Dottori Piccioni, Italy	9.1 g	x	x	x	x
Wheat germ	Laboratorio Dottori Piccioni, Italy	21.8 g	x	x	x	
Cornmeal	(Commercial grade)	18.2 g	x	x	x	
Dry deactivated yeast	Laboratorio Dottori Piccioni, Italy	18.2 g	x			
Apple pulp	Laboratorio Dottori Piccioni, Italy	16.4 g	x	x	x	
Sucrose	(Commercial grade)	36.4 g	x	x	x	x
Ascorbic acid	Sigma-Aldrich	1.8 g	x	x		x
Vanderzant vitamin mix	Brunschwig Chemie, Switzerland	0.8 g	x	x		x
Wesson’s salt	Brunschwig Chemie, Switzerland	1.8 g	x	x		x
Methyl-4-hydroxy benzoate	Sigma-Aldrich	0.5 g	x	x	x	x
Benzoic acid	Merck, Italy	0.5 g	x	x	x	x
Formalin 37%	Merck, Italy	1.0 mL	x			

^a^Quantities are per 1000 mL of diet

### Identification and preparation of yeast species

The yeast species selected for the present study had been previously isolated in our laboratory from *D. suzukii*-infested grapes of the variety ‘Vernatsch’ in 2012. For species identification, DNA was extracted using the NucleoSpin^®^Tissue (Macherey–Nagel, Italy) standard protocol for cultured cells. The partial sequences of 26S rDNA were amplified using the universal primer pair NL1 (5′-GCA TAT CAA TAA GCG GAG GAA AAG-3′)/NL4 (5′-GGT CCG TGT TTC AAG ACG G-3′). PCR products were sequenced with NL1/NL4 primers. The obtained sequences were blasted and aligned using the NCBI database displaying a sequence homology from 99 to 100% with deposited database records. All partial 26S rRNA gene sequences were deposited in GenBank NCBI (Table 2).

**Table 2:** Yeast strains isolated from infested grape

Strain	Phylum	Accession number
*Hanseniaspora uvarum* (LB-NB-1.21)	Ascomycota	KP298009
*Issatchenkia terricola* (LB-NB-2.22)	Ascomycota	KP298010
*Rhodotorula mucilaginosa* (LB-NB-3.5)	Basidiomycota	KP298014
*Candida* sp. (LB-NB-3.3)	Ascomycota	KP298013
*Metschnikowia pulcherrima* (LB-NB-3.2)	Ascomycota	KP298012
*Saccharomycopsis vini* (LB-NB-1.33)	Ascomycota	KP298011

The accession number was deposited in GenBank NCBI

Potato dextrose agar (Merck, Italy) inoculated with yeast-glycerine stock solution was incubated at 28 °C for 4 days. The colonies were then washed off with 0.9% NaCl (Merck, Italy) solution in distilled water. To adjust cell concentrations of the yeast suspensions, the optical density of several dilutions was measured, and the yeast cell number per ml of sterilised saline solution was determined using a Thoma cell counting chamber. The resulting calibration lines for the data pairs of optical density and yeast cell number were used to adjust the concentration of the respective yeast suspension (Anagnostou et al. 2010b).

### Effects of artificial diet components on larval performance

*Drosophila suzukii* larvae from the laboratory population were reared on four different diets: DSCD, DSCD(a), DSCD(b) and DSCD(c) and each of the diets supplemented with 0.04 g of dry live baker’s yeast (*S. cerevisiae*) sprinkled on the surface (area 7.5 cm^2^) for a total of eight treatments. For each replicate, one piece (3 cm length, 2.5 cm width and 0.5 cm height) of each diet treatment was placed in a plastic container (14 cm length, 9 cm width and 4 cm height) and infested with 30 neonate 1st instar larvae per replicate within 24 h after hatching using a thin brush. The experiment was performed three times with three replicates for each diet treatment. A two-way ANOVA with experimental run as the independent factor and development time and survival as dependent variables did not detect any significant differences (*p* > 0.05) between the experimental runs; therefore, the runs were combined (*n* = 9).

Plastic containers with the infested diets were closed with a perforated transparent plastic cover and maintained under standard laboratory conditions. Diet pieces were checked daily to record pupation. As pupae developed, they were removed from the container using a thin brush and placed into separate plastic petri dishes (4 cm diameter) on pieces of wet paper towel to prevent dehydration and checked daily for adult emergence. For each individual that developed on one of the eight different diet treatments, we determined the following life-history traits: (1) larval development time, calculated as the number of days between diet infestation and pupation; (2) pupal development time, calculated as the number of days from pupation to adult emergence; and (3) larval and pupal survival, calculated as the percentage of individuals reaching the next development stage within 30 days.

### Effects of dietary yeast species on larval performance

The low-nutrient diet [DSCD(b)] was used as the standard for testing the dietary effects of different yeast species on the development and survival of the larvae from the laboratory population.

For each yeast treatment, an aliquot of 300 μL of suspension (5 × 10^8^ cells/mL) of the respective yeast species was pipetted onto prepared low-nutrient-diet pieces, fully covering the surface area with the suspension. Additionally, one treatment was prepared with a mix of all yeast cell suspensions (equal cell concentration of each species, 300 µL of cell suspension in total) except that of *S. cerevisiae.*

Three diet pieces per treatment were each infested with 30 neonate larvae from the laboratory population (*n* = 3), and all life-history traits (1–3) were recorded as described above. Additionally, the pupal mass (4) was measured within 24 h after pupation. As the pupae developed, they were removed from the container using a thin brush, rinsed with distilled water to remove residues of the artificial diet and dried for 30 min on a paper towel. The mass of each newly formed pupa was weighed using an analytical balance.

### Effects of nutritional yeast on oviposition performance in a no-choice experiment

In order to obtain sufficient numbers of flies from the laboratory and the wild populations, during a period of 10 days, adults were collected within 24 h after eclosion and maintained in rearing cages provided with 0.5% sucrose solution only. Twenty female and 18 male flies of known age (3–10 days after eclosion) per treatment were then transferred into small experimental cages (50 cm length, 30 cm width and 30 cm height) and fed for 4 days with the respective yeast species (*H. uvarum*, *Issatchenkia terricola*, *Rhodotorula mucilaginosa*, *Candida* sp., *M. pulcherrima*, *Saccharomycopsis vini* and *S. cerevisiae*) cultivated on potato dextrose agar (Merck, Italy) in petri dishes to allow egg production. The agar plates inoculated with yeast were incubated for 5 days at 28 °C until the surface was entirely covered with yeast colonies and then offered to the flies in the experimental cages. For each group of *D. suzukii* flies, adults were exposed to the same yeast treatment in the form of yeast-colonised agar plates during three consecutive test intervals. Yeast agar plates were changed every 3 days to avoid contamination. After 4 days, seven yeast-treated or seven axenic cherry fruits were added to the yeast-colonised agar plates in each experimental cage. Adult flies from the wild population were fed *S. cerevisiae* and additionally tested for an oviposition-stimulating effect of the natural fruit substrate without yeasts on the fruit surface in an axenic treatment because they were not adapted to laboratory rearing conditions.

For each treatment, ripe cherry fruits with stems were inoculated with a yeast suspension (10^8^ cells/mL) of the respective yeast species to stimulate female oviposition on a natural substrate. Seven cherry fruits per treatment were dipped consecutively for 30 s in 0.5% NaOCl, for 30 s in 70% EtOH and for 15 s in sterile dH_2_O to disinfect the surface and then air-dried. For the control treatment, surface-disinfected cherries (axenic fruits) were used. For the yeast treatments, cherries were dipped for 2–3 s each in 15 mL of respective yeast suspension. Thus, seven different treatments with yeast-inoculated cherry fruits and one treatment with axenic cherry fruits were compared. For yeast colonisation, the cherries were maintained for 24 h under standard laboratory conditions before they were put in the experimental cages. Yeast-colonised fruits or axenic fruits were offered to adult flies for a test interval period of 3 days for oviposition, and number of eggs per treatment and per fruit was recorded.

### Statistical analyses

To detect effects of artificial diet components, a general linear model (ANOVA) with larval diet as the independent factor was implemented for larval development time and larval survival. For significant effects, a Tukey’s HSD was conducted. Data on larval development time did not meet the assumption of normality and were log_10_-transformed before analysis. For pupal development time and pupal survival, a nonparametric test was run (Kruskal–Wallis test). Adult oviposition performance was analysed using a general linear model (ANOVA) for repeated measures. Differences between respective yeast species treatments (independent factor) were analysed for consecutive test intervals, and significant effects were detected using the Tukey’s HSD. To meet the assumption of normality, data were log_10_-transformed before analysis. All statistical analyses were conducted using the SPSS statistical software package version 20 (IBM Corp., Released 2011).

## Results

### Effects of artificial diet components on larval performance

**Fig. 1:**
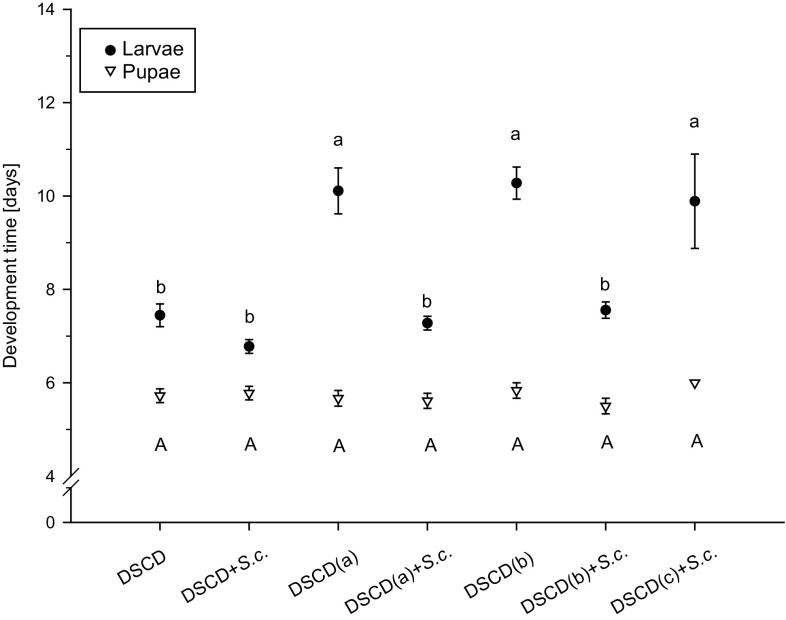
Development of *D. suzukii* on different diet treatments. Development time (mean number of days ± S.E.) of larvae (dark dots, *n* = 9) and pupae (white triangles, *n* = 9) from the laboratory population. No larvae survived on DSCD(c) in the absence of *S. cerevisiae*. Means followed by the same letter (lower case for larvae, upper case for pupae) are not significantly different (development larvae: one-way ANOVA, Tukey’s HSD, *p* < 0.05; development pupae: Kruskal–Wallis test, *p* < 0.05)

The different diet treatments used as larval diet significantly affected the survival (Table 3) and development (Fig. 1) of *D. suzukii* larvae from the laboratory population.

**Table 3:** Average survival (± S.E.) of *D. suzukii* from the laboratory population on different substrate variations

Larval diet	Survival (%) Larvae	Survival (%) Pupae
DSCD	66.67 ± 3.72^ab^	98.83 ± 1.16^a^
DSCD + *S. cerevisiae*	70.37 ± 4.52^a^	98.65 ± 0.68^a^
DSCD(a)	18.15 ± 4.48^e^	95.55 ± 2.93^a^
DSCD(a) + *S. cerevisiae*	52.58 ± 4.36^bc^	96.84 ± 1.69^a^
DSCD(b)	11.83 ± 2.29^e^	98.41 ± 1.59^a^
DSCD(b) + *S. cerevisiae*	45.93 ± 4.07 ^cd^	95.74 ± 2.37^a^
DSCD(c)	0	0
DSCD(c) + *S. cerevisiae*	34.82 ± 3.09^d^	94.78 ± 2.25^a^

Proportion of larvae developing from infestation to pupation, and proportion of pupae developing to adult eclosion (within 30 days)

Values followed by the same lower-case letter within a column are not significantly different from one another (survival larvae: ANOVA, Tukey’s HSD, *p* < 0.05; survival pupae: Kruskal–Wallis test, *p* < 0.05)

Larvae did not survive or survival was significantly reduced when reared on yeast-free diet (*F*_6,42_ = 40.42, *p* < 0.001, *n* = 9). However, larval survival increased distinctly with the addition of *S. cerevisiae*. No larvae survived when fed DSCD(c) in the absence of *S. cerevisiae,* and therefore, their development was not recorded. The highest survival was obtained when larvae were reared on DSCD (66.67 ± 3.72%) and DSCD + S.c. (70.37 ± 4.52%) compared with other diet treatments, whereas pupal survival was apparently unaffected by larval diet (*χ* ^2^ = 2, 32, *df* = 5, *p* = 0.80, *n* = 9).

Regarding larval development, for diet treatments that contained more nutrients (DSCD, DSCD + S.c., DSCD(a) + S.c. and DSCD(b) + S.c.), larval growth increased by approximately 30% compared with other diet treatments (*F*_6,42_ = 19.03, *p* < 0.001, *n* = 9). The diet used for stock maintenance, which also contained the most yeast among all diet treatments, DSCD + S.c., slightly shortened development time of the larvae (6.61 ± 0.1 days) (Fig. 1). By contrast, pupal development did not differ (*χ* ^2^ = 3, 34, *df* = 5, *p* = 0.64, *n* = 9) among the different diets.

### Effects of dietary yeast species on larval performance

**Fig. 2:**
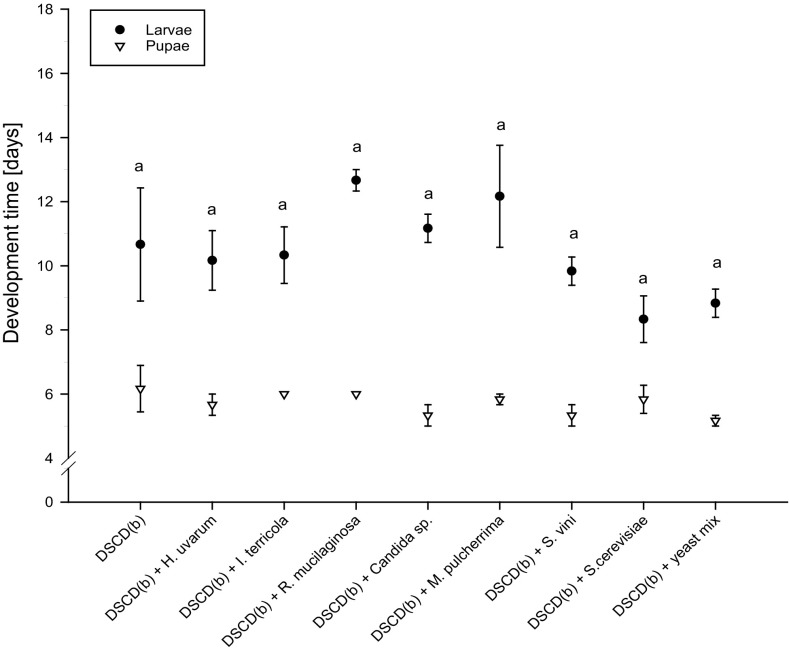
Development of *D. suzukii* on minimal nutrient diet supplemented with aliquots of different yeast species. Development time (mean number of days ± S.E.) of larvae (dark dots, *n* = 3) and pupae (white triangles, *n* = 3). Means followed by the same letter (lower case for larvae, upper case for pupae) are not significantly different (development larvae: one-way ANOVA, Tukey’s HSD, *p* > 0.05; development pupae: Kruskal–Wallis test, *p* < 0.05)

The offered yeast species significantly affected larval survival (*F*_8,18_ = 3.31, *p* = 0.017, *n* = 3; Table 4). The lowest number of larvae survived when reared on the yeast species *M. pulcherrima* (4.44 ± 1.11%), whereas survival was significantly higher when larvae were fed *H. uvarum* (43.33 ± 6.94%) and *Candida* sp. (38.88 ± 2.94%). By contrast, for larval development time, no major differences were detected in the comparison between the low-nutrient diet without yeast supplement [DSCD(b)], which required 10.66 ± 1.76 days, and those diets with different yeast species (Fig. 2). The feeding treatment DSCD(b) supplemented with the yeast species *R. mucilaginosa* (12.66 ± 0.33 days) resulted in the longest development time, whereas larvae fed *S. cerevisiae* developed most rapidly (7.09 ± 0.26 days), but the difference was not significant (*F*_8,18_ = 2.11, *p* = 0.89, *n* = 3). Additionally, pupal development (*df* = 8, *p* = 0.37, *n* = 3) and the percentage of surviving pupae (*df* = 8, *p* = 0.23, *n* = 3) did not differ among the diets with different yeast species. Pupal mass was also not significantly affected by larval diet (*F*_8,18_ = 2.09, *p* = 0.93, *n* = 3), and the range of pupal mass was between 0.85 and 1.30 mg.

**Table 4:** Average survival (± S.E.) of *D. suzukii* on low-nutrient substrate supplemented with aliquots of different yeast species

Larval diet	Survival (%) Larvae	Survival (%) Pupae	Pupal mass (mg)
DSCD(b)	17.77 ± 8.68^ab^	76.66 ± 23.33^a^	0.85 ± 0.07^a^
DSCD(b) + *H. uvarum*	43.33 ± 6.94^b^	78.85 ± 10.27^a^	0.85 ± 0.05^a^
DSCD(b) + *I. terricola*	12.22 ± 5.88^ab^	90.47 ± 9.52^a^	1.30 ± 0.10^a^
DSCD(b) + *R. mucilaginosa*	7.77 ± 2.22^ab^	66.66 ± 19.24^a^	0.94 ± 0.14^a^
DSCD(b) + *Candida* sp.	38.88 ± 2.94^b^	82.30 ± 9.07^a^	0.87 ± 0.05^a^
DSCD(b) + *M. pulcherrima*	4.44 ± 1.11^a^	100 ± 0.00^a^	0.94 ± 0.12^a^
DSCD(b) + *S. vini*	20.00 ± 10.7^ab^	100 ± 0.00^a^	1.01 ± 0.06^a^
DSCD(b) + *S. cerevisiae*	22.22 ± 8.01^ab^	100 ± 0.00^a^	0.90 ± 0.08^a^
DSCD(b) + yeast mix	13.33 ± 5.77^ab^	90.47 ± 9.52^a^	1.02 ± 0.13^a^

Proportion of larvae developing from infestation to pupation, proportion of pupae developing to adult eclosion (within 30 days) and average weight of pupal mass (± S.E.)

Values followed by the same lower-case letter within a column are not significantly different from one another (survival larvae: ANOVA, Tukey’s HSD, *p* < 0.05; survival pupae: Kruskal–Wallis test, *p* < 0.05)

The diet DSCD(b) supplemented with a mix of all single yeast species used in this experiment did not result in any favourable effects on larval or pupal development and survival.

### Effects of nutritional yeast on oviposition

**Fig. 3:**
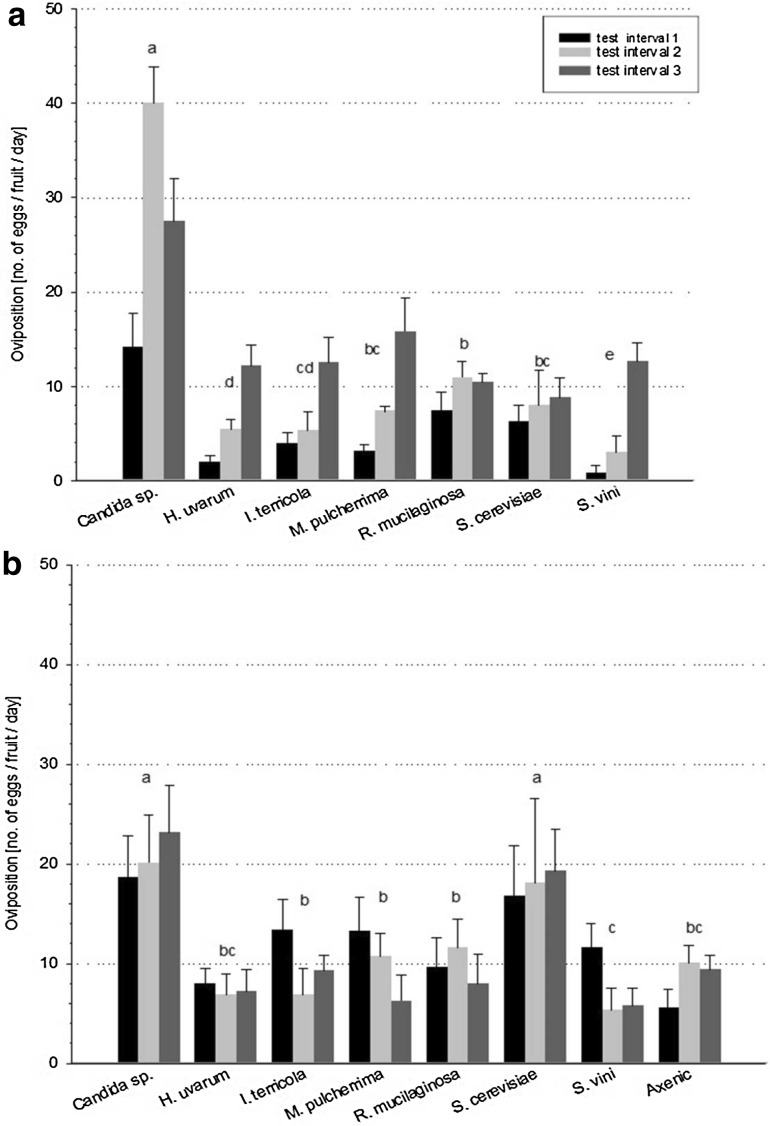
Oviposition of *D. suzukii* females on cherry fruits inoculated with different yeast species. Mean number of eggs/fruit/day (± S.E.) laid by females from **a** the laboratory (*n* = 49) and **b** the wild (*n* = 56) population within three consecutive test intervals. The same yeast species was used to feed the females and to stimulate oviposition on the cherry fruits. Means of the test intervals in the different treatments followed by the same letter are not significantly different (Tukey’s HSD, *p* > 0.05)

Female oviposition was significantly affected by adult nutrition (Fig. 3). In general, oviposition of both the laboratory (*F*_2,84_ = 200.66, *p* < 0.001, *n* = 49) and the wild (*F*_2,96_ = 4.06, *p* = 0.02, *n* = 56) population differed significantly among the three different test intervals. Adult *D. suzukii* females from the laboratory population laid significantly more eggs (treatment: *F*_6,126_ = 79.75, *p* < 0.001, *n* = 49; test interval: *F*_2,126_ = 175.18, *p* < 0.001, *n* = 49; interaction: *F*_12,126_ = 14.92, *p* < 0.001, *n* = 49) on cherry fruits when they had been previously fed with *Candida* sp. (14.19 ± 1.36, 40.07 ± 1.44 and 27.48 ± 1.73 eggs per fruit) in all test intervals (Fig. 3a). Similar results were obtained for the oviposition performance of females from the wild population (Fig. 3b). Females from the wild population laid significantly more eggs (treatment: *F*_7,144_ = 35.53, *p* < 0.001, *n* = 56; test interval: *F*_2,144_ = 4.04, *p* = 0.02, *n* = 56; interaction: *F*_14,144_ = 6.94, *p* < 0.001, *n* = 56) in *Candida* sp. (18.5, 20.0 and 23.1 eggs per fruit) and *S. cerevisiae* treatments (16.7, 18.0 and 19.2 eggs per fruit) than in all other treatments. The number of eggs counted on axenic fruits was significantly lower than that on *S. cerevisiae*-colonised fruits, although females from both populations had been fed previously with *S. cerevisiae.*

## Discussion

We found clear effects of nutritional quality on the life-history traits of *D. suzukii* by modifying the nutrient composition of the rearing substrate. Development of *D. suzukii* larvae was significantly affected by the larval diet. Diet variations that contained more nutrients resulted in a significant increase in larval survival and a significant reduction in larval development time compared with those diets in which nutrient content was lower. Although some diet treatments contained artificial micronutrients or wheat germ as an appropriate protein source for the nutrition of *D. suzukii*, the reduction in development in the absence of yeast indicated a nutritional limitation. Thus, additional evidence is provided by our results that dietary yeast is a primary food source for larval development in *Drosophila* species (Begon 1982; Becher et al. 2012). The amino acid, sterol, vitamin B and fatty acids content is often very low in plant material, and therefore, insect herbivores obtain the nutrients they lack from plant-associated micro-organisms (Vega and Dowd 2004). Indeed, in many insect herbivores, dietary yeast is essential to nutrition not only as a protein source but also for micronutrients such as vitamins, mineral salts and cholesterols that are produced by microbial metabolism (e.g. Sang 1978; Fanson and Taylor 2012).

In addition to the findings of Anagnostou et al. (2010b), Becher et al. (2012) and Buser et al. (2014) on the effects of different yeast species on *D. melanogaster* life-history traits, our results demonstrating the importance of dietary yeast in *D. suzukii* nutrition also suggested a species-specific suitability of yeasts for the development of *D. suzukii*. Therefore, we examined the dietary effects of different host-associated yeast species on the development of *D. suzukii* larvae. In this study, the selected yeasts differed substantially in their suitability for larval development, causing effects on larval survival. The yeast species *H. uvarum* and *Candida* sp. caused a slight beneficial effect on *D. suzukii* survival. Scheidler et al. (2015) also found *H. uvarum* to be the most attractive among other *Drosophila*-associated yeasts when offered in a choice test. By contrast, a distinct decline in larval survival was detected when *D. suzukii* larvae were reared on the yeast species *M. pulcherrima*. Anagnostou et al. (2010b) found similar effects of *M. pulcherrima* on fitness traits of *D. melanogaster* with notably reduced survival rates and longer development times. They also observed that larvae showed no preference for this yeast when offered in a choice test. The absence of suitable nutrients or a disturbance to larval growth by specific metabolic products of the yeast was used to explain their results. By contrast, the larval development time of *D. suzukii* in our experiments was apparently not affected by individual yeast species. However, for other insect species, e.g. the codling moth *Cydia pomonella* (L.), there is some evidence for a mutualistic interaction with *Metschnikowia* yeasts. The yeast *M. andauensis* in larval galleries had a beneficial effect on codling moth larvae by accelerating their development and by reducing mortality (Witzgall et al. 2012).

The variable suitability of a single yeast species for larval nutrition, demonstrating positive or negative effects on larval fitness traits, suggests that the composition of nutrients derived from yeasts is highly heterogeneous. Among different yeast species, the diversity in sugar transport and metabolism mechanisms, intracellular proteins and enzymes for the regulation of different pathways of respiration and fermentation is high (Flores et al. 2000). Thus, a species-rich community of dietary yeast may provide a preferable and balanced supply of nutrients for insect development. The micronutrients provided to insects by individual yeast species may be relevant, in addition to effects on insect development by the mediation of digestive and detoxifying reactions (Vega and Dowd 2004). Rohlfs and Kürschner (2010) reported beneficial effects on larval development from functional yeast metabolites in a species-rich community on mould-infested substrate. To suppress noxious microbes, functional metabolites may be more effective in a community of different species. However, the relevance of species-rich yeast nutrition lies in the specific composition of the respective yeast community (Rohlfs and Kürschner 2010). In our experiment, life-history traits were unaffected by the variation with a yeast mixture, possibly because the composition of yeast species was unsuitable or *M. pulcherrima* was in the mix. Possible negative effects on *D. suzukii* survival could be determined by the exclusion of single yeast species from the diet in further experiments.

Dietary yeast affected female oviposition performance, and significant differences were observed in the number of eggs laid on cherry fruits of the single yeast treatments. Females from both the laboratory and the wild population treated with the yeast *Candida* sp. laid significantly more eggs than the females dedicated to the other yeasts. Increased oviposition activity of females from the wild population was also detected in the *S. cerevisiae* treatment. Although egg maturation in *Drosophila* female flies is dependent on the availability of yeast (Bouletreau-Merle et al. 1978; Powell 1997), detailed information on the suitability of different yeast species for oogenesis is scarce. The findings of Buser et al. (2014) indicate that flies associated with more attractive yeasts display higher female fecundity. Our no-choice oviposition assay with females from the wild population provided further evidence that female flies may be more attracted to cherry fruits inoculated with yeast for oviposition. Females fed *S. cerevisiae* laid significantly more eggs on fruits colonised with that yeast species than on axenic fruits, indicating potential olfactory stimulation for oviposition induced by the yeast on the fruits. This preference of females to oviposit on a substrate optimal for offspring development is consistent with the preference–performance hypothesis (Thompson 1988), which states that the selection of a suitable breeding site by *Drosophila* females is crucial to ensure survival of their larvae. However, similar to the findings of Anagnostou et al. (2010b), in our studies, no distinct correlation was found between the yeast species most suitable for larval development and those favouring adult oviposition, suggesting a combination of both attractiveness of the yeast as a substrate for oviposition and the nutritional contribution of the yeast. Scheirs et al. (2000) propose an explanation for these results and suggest that the oviposition preference of females is for food that is most favourable for their own nutrition. Thus, the yeast that supported the highest numbers of eggs in the present study might be most favourable for *D. suzukii* adult nutrition, i.e. for egg maturation. Based on this strategy, although the yielded progeny might be smaller or slower in their development because of a less suitable breeding site, the benefit of an increase in offspring production to ensure a high population density might be more important.

In conclusion, the results of the present study emphasised the importance of dietary yeast to *D. suzukii* larval development and demonstrated differences in larval survival and female oviposition due to different yeast species. Our findings indicated a strong association of the invasive pest species *D. suzukii* with yeasts. In the literature, yeasts are recognised as a major food source for most *Drosophila* species (Begon 1982), which may show specific relations with various yeast species (Starmer 1981, 1982; Begon 1982; Vacek 1982; Starmer and Fogleman 1986; Starmer et al. 1990; Becher et al. 2012; Chandler et al. 2012; Hamby et al. 2012; Buser et al. 2014). To our knowledge, we are the first to demonstrate the importance of yeasts for the development of the pest species *D. suzukii*.

Based on the results of our study, further detailed research is required to understand the interactions among host plants, microbial flora and pest insect biology and to develop ecologically sound pest control methods. Attractive or nutritionally suitable yeast species as attractants or feeding stimulants could be used in novel behavioural control strategies, including attract-and-kill technologies, against *D. suzukii* (Hamby and Becher 2016; Mori et al. 2017). For example, in organic fruit production, the flies could be lured into specific traps in which they contact spores of an entomopathogenic fungus that are subsequently disseminated through the population. Knight et al. (2016) have previously shown that the yeast species *S. cerevisiae* and *Aureobasidium pullulans* significantly improved the efficacy of particular insecticides when added as a feeding stimulant. However, further research on the efficient use of yeasts in pest control strategies should acquire more detailed information on the biological interaction between insect and yeast.

## Author contributions statement

NB, RZ and EHK conceived and designed the research. NB performed the experiments and analysed data. AG, GI and SS contributed tools and methods. All authors contributed to writing the paper with NB as the lead author.
